# Optimization of a Protocol for the Cryopreservation of Sperm in Pellets for the Common Pheasant (*Phasianus colchicus mongolicus*)

**DOI:** 10.3390/ani11082472

**Published:** 2021-08-23

**Authors:** Annelisse Castillo, Carla Lenzi, Andrea Pirone, Alessandro Baglini, Silvia Cerolini, Nicolaia Iaffaldano, Stefano Sartore, Claudia Russo, Achille Schiavone, Margherita Marzoni Fecia di Cossato

**Affiliations:** 1 Dipartimento di Scienze Veterinarie, Università degli Studi di Torino, Largo Paolo Braccini 2, 10095 Grugliasco, Italy; annelisse.castillogarrido@unito.it (A.C.); stefano.sartore@unito.it (S.S.); 2 Dipartimento di Scienze Veterinarie, Università di Pisa, Viale delle Piagge 2, 56124 Pisa, Italy; carla.lenzi@unipi.it (C.L.); andrea.pirone@unipi.it (A.P.); alessandro.baglini@unipi.it (A.B.); claudia.russo@unipi.it (C.R.); margherita.marzoni@unipi.it (M.M.F.d.C.); 3 Dipartimento di Medicina Veterinaria, Università degli Studi di Milano, Via dell’Università 6, 26900 Lodi, Italy; silvia.cerolini@unimi.it; 4 Dipartimento Agricoltura, Ambiente e Alimenti, Università degli Studi del Molise, Via Francesco De Sanctis, 86100 Campobasso, Italy; nicolaia@unimol.it

**Keywords:** pheasant semen, freezing process, DMA, pellets

## Abstract

**Simple Summary:**

The cryopreservation of avian semen is currently used to preserve species, breeds, and breeding lines. Bird sperm have unique characteristics, and each species responds differently to the freezing–thawing process; in this context, little information about the cryopreservation of the semen of the common pheasant is available. In this study, we tested different parameters at each step of the process of freezing into pellets and thawing with the aim of detecting the least deleterious parameter settings. The pheasant sperm exhibited a high susceptibility to the damage caused by the process of freezing into pellets; however, the survival rate of the sperm was 29%, and the greatest recovered mobility was 22%. The mobility of the sperm was affected by the dilution and the concentration of the dimethylacetamide cryoprotectant; the viability of the sperm was affected by both the equilibration at 5 °C and the dimethylacetamide equilibration. The protocols that caused the least damage to the pheasant sperm were found to be those with higher dilution rates, 10 min of equilibration at 5 °C and equilibration of 6% dimethylacetamide for 1 or 5 min. In the present study, we individualise some applicable parameters for certain critical steps of the freezing–thawing process; however, further investigations are needed in order to improve upon and complete a suitable protocol for the cryopreservation and thawing of pheasant sperm.

**Abstract:**

The sperm of each avian species and breed have unique characteristics that render them more or less susceptible to the freezing–thawing process; therefore, a suitable cryopreservation protocol that is specific for the sperm of each type of bird is needed. In this context, little information about the common pheasant’s sperm is available. Therefore, the aim of this study was to test different parameters at each step of the process of freezing into pellets and thawing to detect the least deleterious parameter settings. Sixteen different protocols were tested by studying two levels in each of the four steps (dilution, equilibration at 5 °C, final dimethylacetamide concentration, and dimethylacetamide equilibration time) comprising the freezing process. The pheasant sperm exhibited a high susceptibility to the damage caused by freezing into pellets; however, the survival of the sperm reached 29%, and the greatest recovered mobility was 22%. The mobility of the sperm was affected by the dilution and the dimethylacetamide concentration, and the viability of the sperm was affected by the equilibration at 5 °C and the dimethylacetamide equilibration. The protocols that caused the least damage to the pheasant sperm were found to be those with higher dilution rates, 10 min of equilibration at 5 °C, and 6% dimethylacetamide equilibrated for 1 or 5 min. In the present study, we individualise some applicable parameters for certain critical steps of the freezing–thawing process; however, further investigations are needed in order to improve upon and complete a suitable protocol for the cryopreservation and thawing of pheasant sperm.

## 1. Introduction

The ability of avian sperm to survive the freezing–thawing process and to remain functional differs not only among species or breeds [1], but even among individuals [2,3,4]. Several factors may contribute to the success of the cryopreservation of sperm, such as the birds’ breeding conditions, the semen collection procedure, the freezing–thawing protocol, etc. [1].

The freezing–thawing process comprises different critical steps, which begin with a diluent and its concentration. The diluent should offer the sperm a suitable environment in terms of pH, osmolality, electrolyte composition, nourishment, protection, etc. in order to face the stresses of the freezing–thawing process [1,2,3,4,5]. The diluent concentration is also crucial; it should provide the cells with enough space and access to oxygen and substrates, as well as space for the metabolic waste [1,5,6]. 

Additional key factors that influence the success of the cryopreservation process are the cryoprotectant, its concentration, its combination with the cooling rate, and its equilibration time [1,4,7]. Cryopreservation is the use of extremely low temperatures to preserve intact and living cells and tissues [8], but very low temperatures cause freezing damage to cells if they are not protected [1,4]. Freezing injuries might be provoked by the freezing of water, which forms ice crystals that are able to pierce the cells, or damage may be caused by changes in the lipid composition of the cell membrane [8,9]. To prevent these negative effects, cells must be protected by a “cryoprotectant”. The cryoprotectant is characterised by its ability to penetrate cells and all parts of the system, thus reducing the amount of ice that can be formed. Another characteristic of a cryoprotectant is that it must have a low toxicity for the cell [8]. The cryoprotectant acts by increasing the total concentration of all solutes in the system, even if numerous barriers (for example, membranes) are encountered that impede the free diffusion of solutes during the penetration into the cells. This situation may cause osmotic fluctuations, resulting in important effects throughout the various steps of the freezing–thawing process [8,9,10].

The next step of the cryopreservation process is the freezing method, which may be slow or more rapid, and the packaging into pellets or straws. Finally, the last parts of the process are the thawing method and temperature of thawing [1]. For all of the factors mentioned above, specific cryopreservation protocols are needed for each species or breed [11]. In birds, several studies have described different protocols for various domestic and non-domestic birds [1,4,12,13,14,15,16]. Among these, semen from a few wild pheasant species was also cryopreserved [17,18]. According to the Convention on International Trade in Endangered Species of Wild Fauna and Flora, nineteen species of the Phasianidae family are included in Appendix I as the most endangered [19]. The greatest ex situ populations of bird species that can breed in captivity are kept by zoos [17]. In this context, sperm cryopreservation technology might be useful; in particular, the common pheasant might be used as a model that can be applied to Phasianidae species. In the past, some in vitro aspects of the freezing–thawing process were studied in our laboratory [20,21,22], in addition to the in vivo response of sperm after being thawed [23,24], the hatchability of pheasant eggs fertilised with thawed sperm [25], and the bird-breeding conditions for collecting high-quality ejaculate [26]. However, for the semen of the common pheasant, there is still very little information regarding its cryopreservation. Therefore, the aim of this study was to test different parameters at the various steps of the freezing–thawing process in order to determine the least deleterious parameter settings for the pheasants’ sperm.

## 2. Materials and Methods

### 2.1. Experimental Design

The model used for the freezing–thawing (F-T) protocol for the pheasant semen had a 2 × 2 × 2 × 2 design: dilution (1:2 or 1:3), equilibration at 5 °C (10′ or 30′), dimethylacetamide (DMA) concentration (6% or 9%), and DMA equilibration time (1′ or 5′). All of the combinations of these factors were applied in the F-T process.

### 2.2. Reagents

All chemicals were purchased from Sigma-Aldrich Chemical Co., Milan, Italy. Accudenz was purchased from Accurate Chemical and Scientific Corp., Westbury, NY, USA.

### 2.3. Birds

Thirty common male pheasants (*Phasianus colchicus mongolicus*) in their first reproductive season were housed in a peaceful location far from crosswalks in open-air aviaries with perches; each male had a 6 m^2^ space [26]. The birds were fed ad libitum on a standard commercial feed (11.51 MJ/kg of M.E., 19% of C.P.). The trial lasted 45 days and began in the middle of April. The males were trained for semen collection for two weeks prior to beginning the trial.

### 2.4. Semen Processing

Semen was collected by using the dorso-abdominal massage technique [27], which was slightly modified to obtain each bird’s spontaneous cloaca eversion and the ejaculation, as described by Castillo et al. [28]. Briefly, two operators were needed. The first operator used both hands to massage and stimulate the bird; the second one collected the ejaculate by means of a pipette. At 3-day intervals, twenty to twenty-five ejaculates of good quality were collected per sampling time. In each week of the trial, one day of semen sampling was set aside for the study (a total of 6 days).

Semen was collected from each male and directly introduced into a collection tube with 50 µL of Lake’s diluent [29]. Immediately thereafter, it was placed inside a portable refrigerator at 18 °C while waiting until all donors were done. Only dense, milky white ejaculates were chosen for analysis and processing, and those with an uncharacteristic colour and/or fluidity were rejected. The ejaculate volume was assessed by weighing the tubes before and after collection (Sartorius BL 150S ± 0.001 g). The semen was pooled, and an aliquot was taken to assess the quality of the fresh semen. The sperm concentration was assessed in duplicate with a Bürker–Türk counting chamber (in 5% formalin and 0.9% NaCl solution) [30]. The percentage of viable sperm was evaluated on 500 cells in triplicate through eosin–nigrosin staining [31]. Viable cells were not stained at all; cells that were considered dead appeared totally or partly stained pink. The percentage of viable sperm was calculated based on the sperm count. Sperm mobility was assessed in triplicate with the Accudenz methodology [32]. The mobility, as opposed to the ability of the sperm to move (motility), is the ability of the cell to penetrate a viscous medium with a temperature of 41 °C, and this penetration was measured with a spectrophotometer at an absorbance of 550 nm [32].

### 2.5. Methodology of Freezing into Pellets

The semen was frozen with the pellet methodology [33]. Sixteen different protocols were tested by combining two variants in each of the four steps (dilution, equilibration at 5 °C, final DMA concentration, and equilibration time with the DMA) comprising the freezing process. The pooled semen was divided into two aliquots and diluted at a ratio of 1:2 or 1:3 (*v/v*) in Lake’s diluent [29] (dilution = D). Diluted pools were then divided into 350 µL aliquots and rapidly cooled to reach a temperature of 5 °C; they were kept in this condition for exactly 10 min or 30 min (equilibration at 5 °C = Eq5 °C). Thereafter, the 5 °C DMA cryoprotectant, which was at a final concentration of 6% or 9% (DMA), was added, mixed manually for one minute in an ice-water bath, and allowed to equilibrate for 1 or 5 min inside the bath (equilibration with DMA = EqDMA). The semen was then frozen by dropping it from a height of 20 cm into a liquid nitrogen bath. The drop volume used was 80 µL. The pellets were grouped by protocol and stored in cryovials containing 6–7 pellets each. The pellets were thawed by placing one pellet at a time onto a hotplate at 50 °C [22].

### 2.6. Thawed Sperm Quality

The qualitative parameters recorded in the frozen–thawed (F-T) sperm were the following: the percentage of viable sperm was evaluated on 100 cells in triplicate through eosin–nigrosin staining [30] and was calculated based on the consideration of the thawed cells as 100%. From the live F-T sperm, normal cells were detected, and their percentage was calculated with respect to the viable F-T cells. Within the portion of live F-T sperm, cells were grouped according to lesions in their head or tail. The head injuries were grouped according to if they were bent, fractured, coiling, swollen/detached, knotted, and lacking a tail. The tail injuries identified were looping, coiling, and lacking a head; these data are reported as the percentage of live F-T sperm. Sperm mobility was assessed by following the same procedure as that reported for the fresh sperm [32]. The mobility is reported as the absorbance units at 550 nm, and the recovered mobility is expressed as the percentage of fresh sperm mobility.

### 2.7. Statistical Analysis

The results are presented as the mean ± standard deviation (SD). All analyses were performed at a significance level of *p* < 0.05 using JMP Statistical Discovery (SAS Institute Inc., Cary, NC, USA, v. 5.0.1.). The percentage data were normalised through √x arcsine transformation. The parameters of the fresh semen obtained on the six collection days were analysed using a one-way ANOVA, followed by Tukey’s test for comparison of means. The following general linear model was used:

Y_ij_ = µ + α_i_ + ε_ij_ (1)

where Y_ij_ is the observation, μ is the overall mean, α_i_ is the fixed effect of the i^th^ day of collection (i = 1 to 6), and ε_ij_ is the random error.

To determine the effects of dilution, Eq5 °C, DMA, and EqDMA on the sperm variables, the data were analysed using a four-factor ANOVA, followed by Tukey’s test for comparison of means. After running the full model, a custom model was set up to exclude all of the interaction terms that were not significant and of no interest. The following general linear model was used:

Y_ijklm_ = µ + α_i_ + β_j_ + γ_k_ + δ_l_ + (αβ)_ij_ + (αβγ)_ijk_ + (αβγδ)_ijkl_ + ε_ijklm_ (2)

where Y_ijklm_ is the dependent sperm variable, μ is the overall mean, α_i_ is the fixed effect of dilution (i = 1:2, 1:3), β_j_ is the fixed effect of Eq5 °C (j = 10′, 30′), γ_k_ is the fixed effect of DMA (k = 6%, 9%), δ_l_ is the fixed effect of EqDMA (l = 1′, 5′), (αβ)_ij_ is the fixed effect of the interaction between dilution and Eq5 °C, (αβγ)_ijk_ is the fixed effect of the interaction between dilution, Eq5 °C, and DMA, (αβγδ)_ijkl_ is the effect of the interaction between dilution, Eq5 °C, DMA, and EqDMA, and ε_ijklm_ is the random error.

## 3. Results

The day of collection did not affect the quality parameters of the fresh semen; the following values were recorded: The mean ejaculation volume was 135 ± 13 µL, the mean sperm concentration was 12.408 ± 1.08 × 10^9^/mL, the mean viability was 91.4 ± 1.98%, and the mean mobility was 0.206 ± 0.0025 absorbance units. Compared to the fresh sperm, the thawed sperm showed a significant loss in both viability (between 79.8% and 98.5%, *p* < 0.01) and mobility (between 85.2% and 95.7%, *p* < 0.01).

Table 1 reports the quality parameters of the frozen pheasant semen according to the effects of the parameters at each step of the freezing procedure. The effects of the two dilution levels were significantly higher in the more diluted samples for both variables—namely, the mobility (*p* < 0.01) and the recovered mobility (*p* < 0.05). The percentage of live sperm was statistically higher when the Eq5 °C lasted 10 min rather than 30 min (*p* < 0.01). A difference due to the cryoprotectant was observed in the mobility, which was more favourable with the lower concentration (6%, *p* < 0.01). A longer EqDMA turned out to be more advantageous for the percentage of live sperm (*p* < 0.01).

The combinations of the two variants in each of the four steps (dilution, Eq5 °C, DMA, and EqDMA) of the F-T process resulted in sixteen different protocols that were tested (Table 2).

Higher percentages of live sperm were mainly observed with higher dilution rates, 10 min of Eq5 °C and 5 min for the EqDMA, and the protocols that gave the best results were characterised by the following parameter levels: 1:2-10′-9%-5′, 1:3-10′-6%, or 9%-5′, 1:3-30′-6%-1′ (*p* < 0.01). The percentage of normal cells was not affected by the protocols. The sperm mobility was higher with the more diluted semen, a Eq5 °C of 10 min, 6% DMA, and an EqDMA of 1 minute, and the protocols that gave the best results were characterised by the following parameter levels: 1:3-10′or 30′-6%-1′, 1:3-10′-6%-5′, 1:3-30′-9%-1′, and 1:2-10′-6%-1′ (*p* < 0.01). A higher recovered mobility was also observed with the higher dilution rate and with the 6% final DMA concentration, and the best results were characterised by the following parameter levels: 1:3-10′or 30′-6%-1′, 1:3-10′-6%-5′, 1:3-30′-9%-1′, 1:2-10′-6%-1′, 1:3-10′or 30′-9%-5′, and 1:2-30′-6%-5′.

Table 3 and Table 4 show the head and tail injuries in the pheasant sperm that were subjected to the freezing–thawing process. A difference was observed in the Eq5 °C step, which showed a higher number of sperm with bent heads for the value of 10 min (Table 3; *p* < 0.01). In addition, the tail injuries were different with the same Eq5 °C, as coiling tails were more frequently observed (Table 4; *p* < 0.05). A higher incidence of looping tails (Table 4; *p* < 0.01) and sperm without tails was observed with 9% DMA (Table 3; *p* < 0.05).

The EqDMA affected the incidence of swollen/detached and knotted heads, and it was less detrimental when using one minute rather than five minutes (Table 3; *p* < 0.05). The EqDMA of one minute affected the occurrence of fractured cells (Table 3; *p* < 0.05). A joint effect of D*Eq5 °C*DMA was observed on the coiling of tails (Table 4; *p* < 0.05), and the combined effect of D*Eq5 °C*DMA*EqDMA was also observed in sperm with no tails (Table 3; *p* < 0.05).

## 4. Discussion

### 4.1. Fresh Semen

The quality of fresh semen is the first element to consider for a more promising performance with sperm that has undergone a freezing–thawing process. The fresh material in this study showed a high viability (91%) compared to that in previous studies of pheasants, which reported mean values of 84–88% [20,21,34]. The sperm mobility is also among the most critical aspects of sperm quality [9], which, in this survey, was in accordance with the findings of previous reports, which indicated absorbances of between 0.180 and 0.320 units [23,34,35]. According to our previous studies, the sperm concentration observed in this trial may be classified as highly concentrated, as evidenced by the values reported throughout the years, which varied from 5 × 10^9^/ mL to 12.5 × 10^9^/ mL [22,23,25,26,36].

### 4.2. The Extender

As expected, the F-T-processed sperm showed a significant loss of viable and mobile cells compared with the fresh sperm. Many factors play key roles throughout the F-T process by acting on the integrity and performance of the sperm [37]. Some of these factors are intrinsic to the sperm, and others are linked to the F-T protocol. The individuation of a suitable F-T protocol for a specific species or breed implicates the testing of different variants and combinations in each step of the F-T protocol. The first question is which diluent to test; Saint-Jalme et al. [17] tested Lake’s diluent [29] in wild pheasant species, and according to these authors, the extender had no effect on the viability or survival rate after the F-T process. Moreover, Tselutin et al. [33] reported high fertility rates in rooster sperm that was exposed to cryopreservation in pellets and a thawing method with a hotplate when employing Lake’s diluent [29]. Therefore, we chose to test Lake’s diluent [29] for the common pheasant. In agreement with Saint Jalme et al. [17], in this study, the diluent had no effect on the viability or the percentage of normal cells. However, the mobility (*p* < 0.01) and the recovered mobility (*p* < 0.05) were affected. Of the two dilution rates tested, the more diluted sperm (1:3) was more favourable, which was in agreement with the data reported previously [20]. The dilution of semen and, thus, the environment surrounding the sperm cells is essential in avoiding the rapid degradation to which the sperm is exposed in vitro due to its intense metabolism and the high proteolytic activity of the seminal enzymes [1,38]. For example, the fertilizing capacity of sperm in chickens is lost in less than one hour, and in guinea fowl, it takes less than half an hour [38]. In addition, a higher dilution rate was found to be associated with a higher fertility rate [39]. In the chicken [40] and in turkeys [41], more diluted sperm was reported to have better sperm quality parameters. On the contrary, other authors reported that lower dilution rates in the Pearl Guinea fowl were more favourable for the viability and maintenance of normal cells that were tested under short-term storage [42], and in chickens, more motile sperm were observed [43].

### 4.3. The Freezing Process and the Cryoprotectant

Sperm is prepared to withstand the thermal changes during the freezing process in the Eq5 °C step [3]. Cooling temperatures and rates may differ according to the species and to the cryoprotectant that is employed [1]. In this study, the cooling rate was chosen based on Tselutin et al. [33]; a decrease of 1 °C every 18 seconds was used, which brought the semen from 18 to 5 °C. The Eq5 °C of ten minutes was more favourable for the viability of the cells, but higher numbers of bent sperm heads and coiling tails were observed. A higher viability was also noted in a previous study with the same Eq5 °C, and, as in the present study, no effects on the mobility or recovered mobility were found [20]. In mammals, reports showed that among the most critical variables influencing the survival of sperm after the F-T process are the cooling and thawing rates [44]. In fact, during the cooling and freezing, the temperature decrease exposes the cells to biological damage, inducing them to adjust to osmotic and thermic changes [9,45,46,47]. The damages that cells may experience can be reversible, such as a temporary injury of the structure or the membrane permeability, or they can be permanent, which is evidenced by a lack of motility [48]. Therefore, to decrease the damage caused by intracellular ice and to regulate the transition induced by temperature variations, an intracellular cryoprotectant is needed [9,48]. However, if the cryoprotectant concentration is too high, it becomes toxic for the cells [9,48]. One of the most widely used cryoprotectants is DMA, which permits one to obtain high fertility rates, especially with pelleted sperm [33,48,49]. In this study, the effect of DMA was less deleterious for the mobility (*p* < 0.01) at a lower concentration (6%). This result was confirmed by previous data that reported higher sperm mobility when using 6% DMA [21]. The viability was not affected by the DMA in this study, while in a previous report, it was higher with 6% DMA [21]. Contrarily, a lower DMA concentration negatively affected the sperm viability in chickens [40], and a higher cryoprotectant concentration induced higher proportions of intact cell membranes [43]. In pheasants, a higher DMA concentration resulted in greater numbers of sperm without tails or with looping tails. The time employed to equilibrate the pheasant sperm with the cryoprotectant influenced the survival of these cells; 5 min was found to be more suitable. In a previous study that tested 5 and 30 min of equilibration with DMA, the period of 5 minutes was also shown to result in higher viability rates [21]; the same result was found in turkeys [41]. In chicken, the 30-minute DMA equilibration positively affected the sperm viability [40]. These differences highlight the complexity of the F-T process, in which many factors are involved; thus, it is necessary to find an equilibrium among all variants for the optimisation of the cryo-processing of pheasant sperm.

### 4.4. The Protocols and the Evaluated Parameters

The sperm’s response to the evaluated parameters depended on the protocol. For example, the highest sperm viability was observed with the least diluted sperm, an Eq5 °C of 10 min, 9% DMA, and an EqDMA of 5 min. The best mobility and recovered mobility were obtained with more diluted sperm, an Eq5 °C of 10 min, 6% DMA, and an EqDMA of 1 or 5 min, or an Eq5 °C of 30 min, 6% DMA, and an EqDMA of 1 minute. Important knowledge has emerged with these results, even though this scenario occurred in vitro. To validate and test the real performance of the sperm that are treated according to specific protocols, an in vivo evaluation is undoubtedly the best approach. However, for example, in turkeys, to evaluate the capacity of toms to sire, when semen traits such as volume, sperm concentration, sperm viability, and membrane integrity were considered, no significant relationships with the offspring produced were observed [50]. Contrarily, sperm mobility has been demonstrated to have a significant correlation with paternity [50,51,52,53]. In fact, sperm mobility has been reported to be predictive of fertility [54,55]. In addition, it was demonstrated that a higher sperm concentration cannot counteract a lower sperm mobility in terms of the fertility rate [53,56], and according to Froman [52], every mobile sperm cell must be motile, but not every motile sperm cell is mobile. According to our experience [25,57], the trait of sperm mobility may reflect AI outcomes in pheasants. Therefore, apparently, the parameters that are the least deleterious to the pheasant sperm that was frozen in pellets are the following: a dilution of 1:3, an Eq5 °C of 10 min, 6% DMA, and an EqDMA of 5 min. However, further studies are needed in order to improve and individualise a complete and suitable protocol for the cryopreservation of pheasant sperm, including its thawing.

## 5. Conclusions

The pheasant sperm exhibited a high susceptibility to the damage caused by the methodology of freezing into pellets; however, the survival of the sperm reached 29%, and the highest level of recovered mobility was 22%.

Dilution and DMA affected the mobility, and the Eq5 °C and EqDMA influenced the sperm viability. The DMA and EqDMA influenced the cell integrity. The protocols that gave the best values exhibited higher dilution rates, an Eq5 °C of 10 min, 6% DMA, and an EqDMA of 1 or 5 min.

Some applicable parameters for certain critical steps of the F-T process were isolated; however, further studies are needed in order to improve and complete a suitable protocol for the F-T process for pheasant sperm.

## Figures and Tables

**Table 1:** Effect on the quality parameters of common pheasant sperm that was frozen using two levels in each step (dilution rate, equilibration at 5 °C, DMA concentration, equilibration time with DMA) of the process of freezing into pellets (means ± SD).

		Viability (%)	Normal (%)	Mobility _Abs ^5^_	Recovered Mobility (%)
D ^1^	1:2	15.4 ± 7.25	29.9 ± 7.26	0.0150 ± 0.007 ^B^	15.0 ± 3.66 ^B^
	1:3	15.2 ± 6.66	29.2 ± 5.93	0.0234 ± 0.012 ^A^	18.0 ± 6.07 ^A^
Eq5 °C ^2^	10	17.2 ± 7.15 ^A^	29.6 ± 6.64	0.0204 ± 0.011	17.4 ± 5.22
	30	12.8 ± 5.64 ^B^	29.4 ± 6.44	0.0180 ± 0.008	15.5 ± 5.20
DMA ^3^	6	15.5 ± 6.71	30.6 ± 6.26	0.0227 ± 0.011 ^a^	17.5 ± 5.51
	9	15.1 ± 7.18	28.1 ± 6.66	0.0157 ± 0.008 ^b^	15.6 ± 4.85
Eq ^4^	1	13.2 ± 6.55 ^B^	28.3 ± 7.64	0.0199 ± 0.011	17.1 ± 5.14
	5	16.7 ± 6.79 ^A^	30.3 ± 5.63	0.0186 ± 0.010	16.3 ± 5.38
Effect					
D		*p* = 0.8850	*p* = 0.9052	*p* = 0.0055	*p* = 0.0213
Eq5 °C		*p* = 0.0038	*p* = 0.8666	*p* = 0.3480	*p* = 0.3832
DMA		*p* = 0.5444	*p* = 0.5094	*p* = 0.0069	*p* = 0.0994
Eq		*p* = 0.0070	*p* = 0.1702	*p* = 0.6182	*p* = 0.6175
D*Eq5 °C		*p* = 0.8046	*p* = 0.8769	*p* = 0.9165	*p* = 0.9856
D*Eq5 °C*DMA		*p* = 0.0625	*p* = 0.6226	*p* = 0.2098	*p* = 0.2443
D*Eq5 °C*DMA*Eq		*p =* 0.5106	*p =* 0.7744	*p =* 0.5194	*p =* 0.5479

^1^ dilution (*v/v*); ^2^ equilibration at 5 °C (minutes); ^3^ dimethylacetamide (%); ^4^ equilibration time with DMA (minutes). ^5^ absorbance units at 550 nm. ^A^^,B^ Means within a column of each step of the freezing process with different superscripts are significantly different (*p* < 0.01). ^a,b^ Means within a column of each step of the freezing process with different superscripts are significantly different (*p* < 0.05).

**Table 2:** Quality parameters of the common pheasant sperm that was cryopreserved according to 16 freezing protocols that differed according to the two levels employed in each step (dilution, equilibration at 5 °C, DMA concentration, equilibration time with DMA) of the freezing process (means ± SD).

D ^1^	Eq5 °C ^2^	DMA ^3^	Eq ^4^	Live (%)	Normal (%)	Mobility _Abs ^5^_	Recovered Mobility (%)
1:2	10	6	1	7.1 ± 3.2 ^D^	22.5 ± 10.6	0.020 ± 0.000 ^ABC^	17.5 ± 0.2 ^abc^
			5	15.4 ± 4.0 ^CD^	33.6 ± 6.8	0.018 ± 0.009 ^C^	15.8 ± 4.4 ^bc^
		9	1	16.8 ± 7.8 ^BC^	26.5 ± 7.0	0.014 ± 0.006^C^	14.2 ± 3.2 ^bc^
			5	28.8 ± 1.6 ^A^	32.6 ± 1.4	0.013 ± 0.005 ^C^	13.8 ± 3.0 ^bc^
	30	6	1	10.0 ± 3.7 ^CD^	26.8 ± 11.9	0.009 ± 0.007 ^C^	11.5 ± 4.7 ^c^
			5	14.6 ± 10.2 ^CD^	34.1 ± 4.9	0.019 ± 0.007 ^BC^	16.5 ± 3.3 ^abc^
		9	1	8.0 ± 0.2 ^D^	23.8 ± 8.8	0.009 ± 0.001 ^C^	12.0 ± 0.9 ^c^
			5	16.3 ± 3.2 ^BCD^	30.3 ± 6.1	0.017 ± 0.008 ^C^	15.5 ± 4.3 ^bc^
1:3	10	6	1	16.4 ± 7.8 ^BCD^	23.8 ± 8.8	0.035 ± 0.010 ^A^	22.3 ± 1.1 ^a^
			5	20.7 ± 5.2 ^AB^	29.7 ± 5.8	0.032 ± 0.010 ^AB^	21.6 ± 3.8 ^ab^
		9	1	10.2 ± 4.4 ^CD^	29.6 ± 12.0	0.015 ± 0.011 ^C^	15.3 ± 6.9 ^c^
			5	19.2 ± 8.1 ^ABC^	26.5 ± 7.0	0.017 ± 0.013 ^C^	16.7 ± 8.0 ^abc^
	30	6	1	17.6 ± 5.7 ^ABC^	32.5 ± 2.2	0.033 ± 0.004 ^AB^	22.1 ± 1.4 ^a^
			5	9.8 ± 4.3 ^D^	28.6 ± 5.4	0.012 ± 0.009 ^C^	11.2 ± 7.7 ^c^
		9	1	9.1 ± 1.3 ^CD^	29.6 ± 13.6	0.022 ± 0.006 ^ABC^	20.0 ± 4.2 ^abc^
			5	11.9 ± 2.2 ^CD^	25.6 ± 5.1	0.017 ± 0.008 ^C^	15.8 ± 3.7 ^abc^

^1^ dilution (*v/v*); ^2^ equilibration at 5 °C (minutes); ^3^ dimethylacetamide (%); ^4^ equilibration time with DMA (minutes). ^5^ absorbance units at 550 nm. ^A–D^ Means within a column of each step of the freezing process with different superscripts are significantly different (*p* < 0.01). ^a–c^ Means within a column of each step of the freezing process with different superscripts are significantly different (*p* < 0.05).

**Table 3:** Head injuries in frozen common pheasant sperm when using the two levels for each step (dilution, cooling rate, DMA concentration, equilibration time with DMA) of the freezing process in pellets and the step influence on the incidence of these damages (means ± SD).

		Bent	Fracture	Coiling	Swo-Det ^5^	Knotted	No Tail
		(%)					
D ^1^	1:2	0.99 ± 1.75	25.0 ± 13.69	0.3 ± 1.17	16.0 ± 9.07	1.5 ± 1.97	1.3 ± 4.31
	1:3	1.31 ± 2.12	24.1 ± 11.60	0.5 ± 2.63	14.2 ± 9.39	0.7 ± 1.43	1.3 ± 5.22
Eq5 °C ^2^	10	1.76 ± 2.28 ^A^	24.0 ± 11.89	0.6 ± 2.76	13.2 ± 7.84	1.0 ± 1.53	0.6 ± 2.23
	30	0.41 ± 1.07 ^B^	25.2 ± 13.34	0.1 ± 0.58	17.3 ± 10.43	1.1 ± 1.97	2.2 ± 6.76
DMA ^3^	6	1.13 ± 1.71	24.0 ± 12.62	0.6 ± 2.71	15.4 ± 10.03	1.1 ± 1.56	0.2 ± 0.95 ^b^
	9	1.22 ± 2.28	25.2 ± 12.46	0.1 ± 0.59	14.5 ± 8.16	1.1 ± 1.95	2.7 ± 7.12 ^a^
EqDMA ^4^	1	1.58 ± 2.69	28.0 ± 15.16 ^a^	0.7 ± 3.15	12.1 ± 9.18 ^b^	0.4 ± 1.01^b^	1.2 ± 5.84
	5	0.90 ± 1.27	22.3 ± 9.96 ^b^	0.3 ± 1.01	16.9 ± 8.86 ^a^	1.5 ± 0.82^a^	1.3 ± 4.10
Effect							
D		*p* = 0.662	*p* = 0.622	*p* = 0.849	*p* = 0.225	*p* = 0.196	*p* = 0.529
Eq5 °C		*p* = 0.004	*p* = 0.750	*p* = 0.487	*p* = 0.236	*p* = 0.971	*p* = 0.159
DMA		*p* = 0.947	*p* = 0.810	*p* = 0.487	*p* = 0.788	*p* = 0.775	*p* = 0.034
EqDMA		*p* = 0.907	*p* = 0.020	*p* = 0.965	*p* = 0.036	*p* = 0.049	*p* = 0.688
D*Eq5 °C		*p* = 0.438	*p* = 0.082	*p* = 0.453	*p* = 0.120	*p* = 0.471	*p* = 0.945
D*Eq5 °C*DMA		*p* = 0.546	*p* = 0.607	*p* = 0.850	*p* = 0.992	*p* = 0.618	*p* = 0.904
D*Eq5 °C*DMA*_Eq_DMA		*p =* 0.514	*p =* 0.303	*p =* 0.268	*p =* 0.206	*p =* 0.469	*p =* 0.015

^1^ dilution (*v/v*); ^2^ equilibration at 5 °C (minutes); ^3^ dimethylacetamide (%); ^4^ equilibration time with DMA (minutes); ^5^ swollen/detached. ^A,B^ Means within a column of each step of the freezing process with different superscripts are significantly different (*p* < 0.01). ^a,b^ Means within a column of each step of the freezing process with different superscripts are significantly different (*p* < 0.05).

**Table 4:** Tail injuries in frozen common pheasant sperm while using two levels in each step (dilution, equilibration at 5 °C, DMA concentration, equilibration time with DMA) of the process of freezing into pellets and the influence of the steps on the incidence of this damage (means ± SD).

		Looping	Coiling	No Head
		(%)		
D ^1^	1:2	10.2 ± 7.30	0.2 ± 0.51	2.5 ± 3.63
	1:3	12.8 ± 8.20	0.3 ± 0.71	2.4 ± 3.07
Eq5 °C ^2^	10	13.6 ± 7.68	0.4 ± 0.78 ^a^	2.8 ± 3.05
	30	9.2 ± 7.51	0.1 ± 0.26 ^b^	2.1 ± 3.61
DMA ^3^	6	10.1 ± 7.96 ^B^	0.3 ± 0.70	2.4 ± 3.30
	9	13.8 ± 7.33 ^A^	0.2 ± 0.52	2.5 ± 3.36
EqDMA ^4^	1	12.3 ± 7.69	0.2 ± 0.47	3.4 ± 3.68
	5	11.2 ± 8.03	0.3 ± 0.29	1.9 ± 2.92
Effect				
D		*p* = 0.262	*p* = 0.844	*p* = 0.571
Eq5 °C		*p* = 0.081	*p* = 0.024	*p* = 0.084
DMA		*p* = 0.008	*p* = 0.844	*p* = 0.456
EqDMA		*p* = 0.980	*p* = 0.291	*p* = 0.103
D*Eq5 °C		*p* = 0.567	*p* = 0.414	*p* = 0.429
D*Eq5 °C*DMA		*p* = 0.940	*p* = 0.024	*p* = 0.700
D*Eq5 °C*DMA*EqDMA		*p* = 0.984	*p* = 0.097	*p* = 0.790

^1^ dilution (*v/v*); ^2^ equilibration at 5 °C (minutes); ^3^ dimethylacetamide (%); ^4^ equilibration time with DMA (minutes). ^A,B^ Means within a column of each step of the freezing process with different superscripts are significantly different (*p* < 0.01). ^a,b^ Means within a column of each step of the freezing process with different superscripts are significantly different (*p* < 0.05).

## Data Availability

The data presented in this study are available on request from the corresponding author.
